# Supplementary material for: Experimental evaluation of a metofluthrin passive emanator against Aedes albopictus
Source: PLoS One. 2022 May 10;17(5):e0267278. doi: 10.1371/journal.pone.0267278 (PMC9089854; doi:10.1371/journal.pone.0267278)
Supplement: S1 File — (DOCX) [file pone.0267278.s001.docx]

## Supporting Information: supplementary tables

## S1 Table. Final Generalized Linear Mixed Models by Outdoor Trials. Impact of treatment outdoors on the response variables: landings and knock downs.

| **Null Hypothesis** | **Model**  **Type** | **Distribution** | **Response**  **Variable** | **Fixed Effects** | **Random Effects** |
| --- | --- | --- | --- | --- | --- |
| The metofluthrin emanator does not impact *Aedes albopictus* feeding behavior outdoors. | GLMM^a^ | Negative Binomial | Landings | Treatment | Day |
| There is no difference in *Aedes albopictus* feeding behavior at 3-8 meters from a 10% metofluthrin emanator outdoors. | GLMM^a^ | Zero-inflated Negative Binomial | Landings | Treatment*Distance, Temperature, Humidity, Wind Speed  Treatment*factored(Distance), Person, Temperature, Humidity, Wind Speed | Day/Trial, Person  Day/Trial |
| The age of the metofluthrin emanator does not impact *Aedes albopictus* feeding behavior outdoors. | GAM^b^ |  | Landings | Emanator Age |  |

^a^GLMM = Generalized Linear Mixed Model

^b^GAM = Generalized Additive Model

## S2 Table. Final Generalized Linear Mixed Models by Indoor Trials. Impact of treatment indoors on the response variables: landings and knock downs.

| **Null Hypothesis** | **Model**  **Type** | **Distribution** | **Response**  **Variable** | **Fixed Effects** | **Random**  **Effects** |
| --- | --- | --- | --- | --- | --- |
| The 10% metofluthrin emanator does not impact *Aedes albopictus* feeding behavior indoors. | GLMM^a^ | Negative Binomial | Landings | Treatment, Temperature, Humidity, Wind Speed | Day/  Trial |
| There is no difference in *Aedes albopictus* feeding behavior at 1-3 meters from a metofluthrin emanator indoors | GLMM^a^ | Negative Binomial | Landings | Treatment*Time + Distance + Number of Mosquitoes  Treatment*Distance + Number of Mosquitoes | Day, Person  Day, Person |
|  |  | Binomial | Knock downs | Treatment  Distance | Day  Day |
| The metofluthrin emanator does not impact *Aedes albopictus* mortality. | CPHRM^b^ |  | Mortality | Treatment*factored( Distance) + cluster(Group) + Day  Distance + cluster(Group) + Day |  |
| The age of the metofluthrin emanator does not impact *Aedes albopictus* feeding behavior indoors. | GAM^c^ |  | Landings  Knock downs | Emanator Age  Emanator Age |  |

The model assessing the effect of distance from the emanator on mosquito landing used a subset of the original data which included only landings from mosquitoes exposed to a treatment for 60 minutes. Models assessing the effect of distance from the emanator on mosquito knock downs and mortality used a subset of the original data which included only mosquitoes exposed to the emanator.

^a^GLMM = Generalized Linear Mixed Model

^b^CPHR = Cox Proportional Hazards Regression Model

^c^GAM = Generalized Additive Model
