# Supplementary material for: Experimental evaluation of a metofluthrin passive emanator against Aedes albopictus
Source: PLoS One. 2022 May 10;17(5):e0267278. doi: 10.1371/journal.pone.0267278 (PMC9089854; doi:10.1371/journal.pone.0267278)

PLOS ONE

Experimental evaluation of a metofluthrin passive emanator against Aedes albopictus

--Manuscript Draft--

**Manuscript Number:**	PONE-D-21-25159
**Article Type:**	Research Article
**Full Title:**	Experimental evaluation of a metofluthrin passive emanator against Aedes albopictus
**Short Title:**	Metofluthrin emanators against Aedes albopictus
**Corresponding Author:**	Gonzalo M Vazquez-Prokopec, Ph.D.; M.Sc.<br>Emory University<br>Atlanta, GA UNITED STATES
**Keywords:**	Vector control, mosquito, pyrethroid, spatial repellent
**Abstract:**	Background: With the recent occurrence of locally transmitted Aedes -borne viruses in the continental United States and Europe, and a lack of effective vaccines, new approaches to control Aedes aegypti and Aedes albopictus are needed . In sub-tropical urban settings in the US, Ae. albopictus is a dominant nuisance and arbovirus vector species. Unfortunately, the vector control toolbox against Ae. albopictus is not as well developed as for Ae. aegypti . Here, we evaluate the efficacy, longevity, and range of protectiveness of a novel passive metofluthrin emanator (10% active ingredient in a polyethylene mesh) against Ae. albopictus indoors and outdoors. Methods: Four studies were conducted comparing the presence of the metofluthrin emanator to a control lacking emanator with interest in quantifying efficacy by human landing counts. Studies evaluated the effect of an emanator at varying distances from one or more human volunteers indoors and outdoors. Efficacy of emanators over time since activation was also evaluated. Results: Mixed-effects models determined that sitting in close proximity to an emanator reduced landings by 89.5% outdoors and by 74.6% indoors. The emanator was determined protective when located immediately next to a human volunteer outdoors but not uniformly protective when located further away. The emanator was protective at all tested distances from the device indoors. Mortality of mosquitoes exposed to metofluthrin emanators was ~2x higher than those who were not exposed in indoor conditions. Finally, a Generalized Additive Model determined that emanators used continuously outdoors lost their effect after 2.5 weeks and stopped inducing paralysis in mosquitoes after 3.8 weeks of use. Conclusions: We show strong and lasting efficacy of 10% metofluthrin emanators against field Ae. albopictus both in indoor and outdoor conditions. Metofluthrin emanators can protect people from Ae. albopictus bites, representing a viable option for reducing human-mosquito contacts at home and beyond.
**Order of Authors:**	Olivia Zarella
	Uche Ekowmadu
	Yamila Romer
	Oscar D Kirstein
	Azael Che-Mendoza
	Gabriela González-Olvera
	Pablo Manrique-Saide
	Gregor Devine
	Gonzalo M Vazquez-Prokopec, Ph.D.; M.Sc.
**Additional Information:**	
**Question**	**Response**
**Financial Disclosure**<br><br>Enter a financial disclosure statement that	This study was supported by a grant from USAID (AID-OAA-F-16-00094; Devine, PI). The funders had no role in study design, data collection and analysis, decision to publish, or preparation of the manuscript.

Powered by Editorial Manager® and ProduXion Manager® from Aries Systems Corporation

describes the sources of funding for the work included in this submission. Review the submission guidelines for detailed requirements. View published research articles from *PLOS ONE* for specific examples.<br><br>This statement is required for submission and **will appear in the published article** if the submission is accepted. Please make sure it is accurate.<br><br>**Unfunded studies**<br>Enter: *The author(s) received no specific funding for this work.*<br><br>**Funded studies**<br>Enter a statement with the following details:<br>• Initials of the authors who received each award<br>• Grant numbers awarded to each author<br>• The full name of each funder<br>• URL of each funder website<br>• Did the sponsors or funders play any role in the study design, data collection and analysis, decision to publish, or preparation of the manuscript?<br>• **NO** - Include this sentence at the end of your statement: *The funders had no role in study design, data collection and analysis, decision to publish, or preparation of the manuscript.*<br>• **YES** - Specify the role(s) played.<br><br>* typeset	
**Competing Interests**<br><br>Use the instructions below to enter a competing interest statement for this submission. On behalf of all authors, disclose any competing interests that could be perceived to bias this work—acknowledging all financial support and any other relevant financial or non-financial competing interests.<br><br>This statement is required for submission and **will appear in the published article** if the submission is accepted. Please make sure it is accurate and that any funding	The authors declare that they have no competing interests.

sources listed in your Funding Information later in the submission form are also declared in your Financial Disclosure statement.<br><br>View published research articles from *PLOS ONE* for specific examples.<br><br>**NO authors have competing interests**<br><br>Enter: *The authors have declared that no competing interests exist.*<br><br>**Authors with competing interests**<br><br>Enter competing interest details beginning with this statement:<br><br>*I have read the journal's policy and the authors of this manuscript have the following competing interests: [insert competing interests here]*<br><br>* typeset	
**Ethics Statement**<br><br>Enter an ethics statement for this submission. This statement is required if the study involved:<br><br>• Human participants<br>• Human specimens or tissue<br>• Vertebrate animals or cephalopods<br>• Vertebrate embryos or tissues<br>• Field research<br><br>Write "N/A" if the submission does not require an ethics statement.<br><br>General guidance is provided below. Consult the submission guidelines for detailed instructions. **Make sure that all information entered here is included in the Methods section of the manuscript.**	N/A

**Format for specific study types**<br><br>**Human Subject Research (involving human participants and/or tissue)**<br>• Give the name of the institutional review board or ethics committee that approved the study<br>• Include the approval number and/or a statement indicating approval of this research<br>• Indicate the form of consent obtained (written/oral) or the reason that consent was not obtained (e.g. the data were analyzed anonymously)<br><br>**Animal Research (involving vertebrate animals, embryos or tissues)**<br>• Provide the name of the Institutional Animal Care and Use Committee (IACUC) or other relevant ethics board that reviewed the study protocol, and indicate whether they approved this research or granted a formal waiver of ethical approval<br>• Include an approval number if one was obtained<br>• If the study involved *non-human primates*, add *additional details* about animal welfare and steps taken to ameliorate suffering<br>• If anesthesia, euthanasia, or any kind of animal sacrifice is part of the study, include briefly which substances and/or methods were applied<br><br>**Field Research**<br><br>Include the following details if this study involves the collection of plant, animal, or other materials from a natural setting:<br>• Field permit number<br>• Name of the institution or relevant body that granted permission	
**Data Availability**<br><br>Authors are required to make all data underlying the findings described fully available, without restriction, and from the time of publication. PLOS allows rare exceptions to address legal and ethical concerns. See the PLOS Data Policy and FAQ for detailed information.	Yes - all data are fully available without restriction

Powered by Editorial Manager® and ProduXion Manager® from Aries Systems Corporation

A Data Availability Statement describing where the data can be found is required at submission. Your answers to this question constitute the Data Availability Statement and **will be published in the article**, if accepted.<br><br>**Important:** Stating 'data available on request from the author' is not sufficient. If your data are only available upon request, select 'No' for the first question and explain your exceptional situation in the text box.<br><br>Do the authors confirm that all data underlying the findings described in their manuscript are fully available without restriction?	
**Describe where the data may be found in full sentences. If you are copying our sample text, replace any instances of XXX with the appropriate details.**<br><br>• If the data are **held or will be held in a public repository**, include URLs, accession numbers or DOIs. If this information will only be available after acceptance, indicate this by ticking the box below. For example: *All XXX files are available from the XXX database (accession number(s) XXX, XXX.).*<br>• If the data are all contained **within the manuscript and/or Supporting Information files**, enter the following: *All relevant data are within the manuscript and its Supporting Information files.*<br>• If neither of these applies but you are able to provide **details of access elsewhere**, with or without limitations, please do so. For example:<br><br>*Data cannot be shared publicly because of [XXX]. Data are available from the XXX Institutional Data Access / Ethics Committee (contact via XXX) for researchers who meet the criteria for access to confidential data.*<br><br>*The data underlying the results presented in the study are available from (include the name of the third party*	Emanators supplied by Sumitomo Chemical Company Ltd. Data supporting the conclusions of this article are included within the article. Data generated or analyzed during the present study are publicly available from: Vazquez-Prokopec, Gonzalo (2021), "Experimental evaluation of a metofluthrin passive emanator against Aedes albopictus", Mendeley Data, V1, doi: 10.17632/dkdc8642cv.1[embargoed until publication].

and contact information or URL).<br>• This text is appropriate if the data are owned by a third party and authors do not have permission to share the data.<br><br>* typeset	
Additional data availability information:	Tick here if the URLs/accession numbers/DOIs will be available only after acceptance of the manuscript for publication so that we can ensure their inclusion before publication.

Powered by Editorial Manager® and ProduXion Manager® from Aries Systems Corporation

manuscript Click here to access/download;Manuscript;ZARELLA_etal_metofluthrin

Experimental evaluation of a metofluthrin passive emanator against *Aedes albopictus*

Zarella O[1] , Ekowmadu U, Romer Y [1], Kirstein O[1], Che-Mendoza, A[2], Gonzalez-Olvera G[2], Manrique-Saide P[2], Devine G[3], Vazquez-Prokopec GM[1*]

[1]Department of Environmental Sciences, Emory University, Atlanta, GA, USA.

[2]Unidad Colaborativa de Bioensayos Entomológicos, Campus de Ciencias. Biológicas y Agropecuarias, Universidad Autónoma de Yucatán, Mérida, Yucatán, México.

[3]Mosquito Control Laboratory, QIMR Berghofer Medical Research Institute, Brisbane, Queensland, Australia.

[#a]Current Address: Department of Environmental Sciences, Emory University, 400 Dowman Drive, Math and Science Center, 5th floor, Suite E530. Atlanta, GA, 30322, USA.

*Corresponding Author

Email: gmvazqu@emory.edu

Abstract

Background: With the recent occurrence of locally transmitted *Aedes*-borne viruses in the continental United States and Europe, and a lack of effective vaccines, new approaches to control *Aedes aegypti* and *Aedes albopictus* are needed. In sub-tropical urban settings in the US, *Ae. albopictus* is a dominant nuisance and arbovirus vector species. Unfortunately, the vector control toolbox against *Ae. albopictus* is not as well developed as for *Ae. aegypti*. Here, we evaluate the efficacy, longevity, and range of protectiveness of a novel passive metofluthrin emanator (10% active ingredient in a polyethylene mesh) against *Ae. albopictus* indoors and outdoors. Methods: Four studies were conducted comparing the presence of the metofluthrin emanator to a control lacking emanator with interest in quantifying efficacy by human landing counts. Studies evaluated the effect of an emanator at varying distances from one or more human volunteers indoors and outdoors. Efficacy of emanators over time since activation was also evaluated. Results: Mixed-effects models determined that sitting in close proximity to an emanator reduced landings by 89.5% outdoors and by 74.6% indoors. The emanator was determined protective when located immediately next to a human volunteer outdoors but not uniformly protective when located further away. The emanator was protective at all tested distances from the device indoors. Mortality of mosquitoes exposed to metofluthrin emanators was ~2x higher than those who were not exposed in indoor conditions. Finally, a Generalized Additive Model determined that emanators used continuously outdoors lost their effect after 2.5 weeks and stopped inducing paralysis in mosquitoes after 3.8 weeks of use. Conclusions: We show strong and lasting efficacy of 10% metofluthrin emanators against field *Ae. albopictus* both in indoor and outdoor conditions. Metofluthrin emanators can protect people from *Ae.*

albopictus bites, representing a viable option for reducing human-mosquito contacts at home and beyond.

Introduction

The rapid expansion and dramatic global increase in the burden of dengue, Zika and Chikungunya viruses constitutes a major public health problem in tropical and subtropical urban areas [1-4]. Transmitted primarily by *Ae. aegypti* and, to a lesser extent, *Ae. albopictus*, control of these viruses depends primarily on health education and vector control, as no effective vaccine or prophylactic measure is yet available [1,5]. While most of Europe and the continental US have been free of local *Aedes*-borne virus transmission for decades, the last twenty years have seen a rapid increase in locally acquired cases. Traditionally affecting U.S. territories, such as Hawaii, the Virgin Islands, and Puerto Rico [6-8], dengue outbreaks have very frequently occurred in Florida [9] and Texas [10, 11]. Later, outbreaks of chikungunya [12] and Zika [13] in Florida, pointed out the potential role of *Ae. albopictus* in enhancing local transmission [14, 15] in areas where both *Ae. aegypti* and *Ae. albopictus* overlap [16]. Recent outbreaks in Europe and other parts of the world have incriminated *Ae. albopictus* as the main vector [17]. *Ae. albopictus* was associated with significant outbreaks, including the 2005-2006 chikungunya outbreak on the island of Réunion [18] or the 2017 chikungunya outbreak in the Lazio and Calabria regions of Italy [19]. With an increasing number of epidemics linked to *Ae. albopictus*, future control efforts should aim to target more aggressively this vector in areas prone to local arbovirus transmission.

Unfortunately, the knowledge base supporting the effectiveness of available control tools for *Ae. albopictus* control is less developed than for *Ae. aegypti*. Differences among species in spatial distribution, habitat use within cities, and adult female densities indoors and outdoors, human biting rate [20-24] may limit the effectiveness of methods known to impact *Ae. aegypti* on *Ae. albopictus*. For instance, while outdoor barrier spraying may not be effective against *Ae. aegypti*, it is the mainstay of *Ae. albopictus* control in the Torres Strait, Australia, due to its outdoor resting behaviour [25].

Volatile pyrethroids such as transfluthrin and metofluthrin have potential for the control of *Ae. aegypti* when incorporated into passive emanators (insecticide-treated materials that slowly release the active ingredient [a.i.] into the air, without the need for power or heat). These chemicals are captured under the “spatial repellent” paradigm and exert a variety of dose-dependent lethal, repellent and confusant effects on a variety of insects [26, 27]. Under laboratory conditions, metofluthrin is effective (in toxicity and biting inhibition) to *Ae. albopictus* [28]. When used as a mosquito coil and compared to other commonly used volatile pyrethroid a.i. such as transfluthrin and d-allethrin under laboratory conditions, metofluthrin had greater chemical stability [29] and produced the highest *Ae. albopictus* mortality rate (> 80%) [30]. A manufactured passive emanator consisting of a fan-type paper device with a non-heated metofluthrin formula was highly effective against *Ae. albopictus*, reducing biting by 92-97%, but with its effectiveness rapidly decreasing after 48 hours due to the rapid loss of a.i. from the paper matrix [31]. An engineered alternative to this passive emanator, consisting of a resin formulation containing 4.4% metofluthrin, has proven more durable and effective at volatilizing the a.i. compared to paper or other carriers [29]. More recently, a prototype passive emanator containing 10% metofluthrin w/w (SumiOne®, Sumitomo Chemical) has been extensively tested against *Ae.*

aegypti, showing significant impact on indoor abundance and biting [26]. The device consists of an impregnated net housed in a rectangular plastic cartridge designed to be hung indoors using an integral hook. Under semi-field conditions, exposure to the emanator for one hour reduced landing behavior for pyrethroid-susceptible *Ae. aegypti* mosquitos from 32-46 landings to zero [32]. Also, 80-90% of mosquitos were knocked down during that time. An entomological randomized field trial conducted in Ticul, Mexico, found that placing emanators at a rate of one per room per house led to a reduction in indoor female *Ae. aegypti* density of 70% and of >90% reduction in landings compared to houses without emanators [33].

In contrast to the increasing volume of work on the effectiveness of volatile pyrethroids against *Ae aegypti* indoors, there remains a lack of empirical study on their impact indoors or outdoors on *Ae. albopictus* mosquitoes. This study evaluated the efficacy of a 10% w/w/ metofluthrin emanator against pyrethroid susceptible *Ae. albopictus* populations from Southeastern U.S. Specifically, we quantified the impact of emanators on landing behavior, mortality and repellency of *Ae. albopictus* upon exposure to the device in laboratory, semi-natural, and field conditions.

Methods

Study design

The study involved four independent evaluations that occurred either indoors or outdoors depending to the specific hypothesis to be tested. Indoors testing occurred inside a

bedroom from a house located near Emory University. Field testing was conducted in Baker Woodlands, located within Emory University campus in Atlanta, GA (33°47'20.5 "N, 84°19'34.3 "W), a landscape densely populated with *Ae. albopictus*. The human landing count (HLC) method is the gold standard for determining human-mosquito contact rates [34] and was used to quantify the efficacy of the emanators. For our study, a volunteer sat in a camping chair wearing a bug-proof jacket, hood and long socks. The skin on their legs from knees to ankles or arm from hand to elbow (depending on the evaluation) was exposed. During data collection, the technician prevented mosquito biting by brushing away mosquitos immediately after they landed (33). Each landing was counted for a 5 minute period. For experiments in tents and bedroom, an assessment of Knock-down was also made by counting mosquitos exhibiting unusual behavior of being "knocked out" of the air due to paralysis from exposure to the insecticide; representing the mosquito's inability to stand, fly, or take-off [35, 36].

Study 1: Testing the short-distance effect of emanators on Ae. albopictus landing behavior outdoors

To test whether close proximity to an emanator impacts *Ae. albopictus* landing behavior outdoors, a field-trial was designed and applied on four separate days from June 2017 to July 2017. The field-trial consisted of four HLC collections per day (2 metofluthrin treatment and 2 control). A total of 8 tests were conducted per arm, all during the morning, and spaced 1 h apart (Fig 1A). Test treatments were randomized across the 5 min assessments and trials occurred during the summer on days with no rain. Before each trial, the technician sat with unexposed skin for 5 min to acclimate and attract mosquitoes in the area.

Fig 1. Diagram of different studies conducted to evaluate the efficacy of metofluthrin emanators on *Ae. albopictus*

(A) Metofluthrin emanators were hung outdoors on top of a 1 m stick (A) and used to test their effect on human landing counts in the immediate vicinity (<1m distance). (B) Emanators were hung at increasing distances from it up to 8 meters. (C) An outdoor tent experiment was set outdoors to evaluate the impact of emanators on mosquito entry and landing counts. (D) An indoor evaluation of the impact of emanators on *Ae. albopictus* landing counts and knock down was conducted inside a bedroom and placing mosquitoes in bugdorms at 1m and 3m from the emanator.

Study 2. Testing emanator range of activity on *Aedes albopictus* outdoors

Study 1 provided the efficacy of emanators at immediate distance (<1m). To determine the distance over which the emanators were effective outdoors, we examined *Ae. albopictus* landing behavior within a 3-8m radius of the emanator. The field-trial was conducted in the morning, over nine days from August to September 2018. Two locations in Baker Woodlands with similar vegetation and *Ae. albopictus* characteristics, 20m apart, were selected. Each day, a trial consisting of a matched control (no emanator) and treatment (emanator) was conducted in a pair of distances from 3 to 8 meters selected at random. We tested efficacy at 3, 4, 5, 6, 7 and 8 m from the emanator. The same distances were tested in each location, and the assignment of treatment to each location was also selected at random. Four replicates for each distance in each arm (treatment and control) were conducted. Two collectors participated in this study, randomized between treatments and locations. HLC quantification occurred as above. Factors for analysis considered for each test included

cardinal direction, distance, time of day of each test, collection period, HLC, identity of the observer, temperature, humidity, and wind speed and direction.

Study 3: Testing the effect of metofluthrin emanators on entry behavior

To test whether metofluthrin emanators impact *Ae. albopictus* entry and landing behavior inside tents, a trial was conducted outdoors during the morning over four days from August to September 2018. Two four-person tents (Coleman Sundome, Coleman Company Inc.) were set ~20 m apart, with a seated observer inside to assess HLC and a white tarp placed on the floor to assess knock-down (Fig 1C). Tent treatments and locations were randomized between tests and two observers were randomly assigned to each tent. Two 10-minute collections were made for each treatment instead of the 5 min HLCs from before to give more time mosquitoes to enter the tents. Before data collection, a metofluthrin emanator was hung from the ceiling of the designated intervention tent, above the seated volunteer. The tent entrance was closed for ten minutes with one volunteer inside. After this 10-minute acclimation period, the volunteer opened the tent to allow mosquito host-seeking and entry and conducted a 10-minute HLC collection. Variables collected included time, tent location, temperature, humidity, and wind direction and speed.

Study 4. Testing emanator longevity and range of activity indoors

To test how the distance and age of the emanator could affect *Ae. albopictus* landing behavior indoors, we conducted experiments inside a bedroom between October and December 2017. Impacts on landing behavior and knock-down were tested at distances of 1 and 3 meters from the emanator (to capture the extent of the room). We also looked at the effective longevity of the emanators by repeating experiments at weekly intervals and up to 5 weeks.

We followed the design described by Darbro et al. [37] by conducting experiments in a bedroom with dimensions 3.7 m x 3.0 m x 2.4 m. A group of 10 emanators was used throughout the trial and were aged indoors in a ventilated room. Each weekly assessment period, a randomly chosen emanator was used against *Ae. albopictus* female mosquitos raised from F0 eggs collected in Baker Woodlands. The weekly treatments included four levels: untreated control at 1 m from the emanator, untreated control at 3 m, emanator exposed at 1 m, emanator exposed at 3 m. For each treatment, three bugdorm cages (30 cm x 30 cm x 30 cm) containing ten mosquitoes each were placed at the specified location. A similar number of mosquitoes were kept in the laboratory and was used to determine if transport to the houses in a Styrofoam cooler impacted survival. Before each test, mosquitos were acclimated to the temperature of the room for 30 minutes.

We ran the evaluations at the time of opening the emanator (t= 0) and then weekly up to week 5. At every time, we quantified mosquito landings on an exposed arm for a total of 2 minutes and knock-down after a 30 min exposure to the emanator. Control trials were conducted before exposure trials to prevent build-up of the active ingredient in the room. To assess mortality, mosquitos were transferred back to the lab after the completion of each exposure trial and remained in an incubator at 26°C and 80% humidity to assess long-term effects of exposure to metofluthrin. Daily mortality was recorded until 100% of mosquitoes died in both emanator and control treatments.

Mosquito rearing

Mosquitoes used in the study derived from eggs collected in Baker Woodlands and were reared in the insectary of the Department of Environmental Sciences, Emory

University. Briefly, adults and larvae were maintained at 25 ± 5º C and 80 ± 10% humidity, with a 12:12 (L:D) h photoperiod. Larvae were fed with yeast and liver powder (MP Biomedicals) and adults with cotton soaked in 10% sugar solution. Adults of 5 to 7 days from emergence were sugar starved for 24 hours before being used in the experiment. Mosquito susceptibility to major insecticide classes was tested by CDC bottle bioassay [38].

Statistical analysis

Generalized linear models were used for most analysis and can be viewed in S1 and S2 Tables. For HLC, negative binomial generalized linear mixed models (GLMM) were implemented, whereas for studies evaluating emanator longevity a Cox Proportional-Hazards Regression Model (CPHRM) was applied to quantify the probability of mosquito death occurring per day post exposure. Inclusion or exclusion of exploratory variables were determined by using the Akaike Information Criterion (AIC) which compared the model with and without the variable. A summary of the final GLMM model used to assess each hypothesis is found in S1 and S2 Tables. For treatments showing a significant reduction in HLC, we estimated the emanator's efficacy, as 1 - Incidence Rate Ratios (IRR). This metric, bound between 0 and 1, shows the proportional reduction in a metric compared to the control. All analyses were performed in the R statistical software (version 4.0.3) using the package mgcv.

Results

Ae. albopictus susceptibility to insecticides

At the diagnostic time (30 min for pyrethroids, 45 min for DDT), *Ae. albopictus* from the field (and used in lab experiments) evidenced a mortality of 98.5% for permethrin (diagnostic dose, 15 µg/ml), 100% for deltamethrin (diagnostic dose, 10 µg/ml), and 97% for DDT (diagnostic dose, 75 µg/ml). At 90 minutes post-exposure, all mosquitoes were found dead irrespective of the insecticide. Mortality in the controls was 0% for all insecticides and times.

Study 1: Testing the short-distance effect of emanators on Ae. albopictus landing behavior outdoors

We recorded 932 landing counts over four data collection days with 9.0% (84/932) of landings occurring in the presence of an emanator and 91.0% (846/932) of landings occurring during control periods. The mean landing count of the control was at least 5.3 times the mean landing count of the emanator (Fig 2A), with a statistically significant 89.5% reduction in landing counts in the presence of an emanator, Table 1.

Fig 2. Mean landing counts of each treatment by date and distance to the emanator in outdoors trials.

(A) In Study 1, the effect of the emanator on landing counts when placed next to a human volunteer was assessed (B) In Study 2, the effect of the emanator when placed at variable

distances from a human volunteer was measured. (C) In Study 3, the effect of the emanator when placed inside a tent with a human volunteer sitting next to it was tested.

Table 1. Parameter estimates of *Aedes albopictus* HLC post-exposure to an emanator placed next to human outdoors.

Parameter	Value	Standard Error	Z-value	P-value	1-IRR
Intercept	4.5404	0.0809	56.1	**<0.001**	
Treatment (Emanator)	-2.2555	0.251	-8.99	**<0.001**	0.895

In parenthesis is listed the variable used as baseline for effect estimation. For treatments showing a significant reduction in HLC, we estimated the efficacy, as 1 - Incidence Rate Ratios (IRR). Bolded p-values are less than 0.05 and statistically significant. ~~For treatments showing a significant reduction in HLC, we estimated the efficacy, as 1 - Incidence Rate Ratios (IRR)~~.

Study 2. Testing effective distance of emanator activity on Ae. albopictus outdoors

We recorded 1,731 landing counts over the course of nine data collection days with 46.3% (801/1731) of landings occurring in the presence of an emanator and 53.7% (930/1,731) during control periods (Fig 2B*).* In a GLMM including distance as a continuous factor and controlling for environmental variables, location had no significant effect on *Ae. albopictus* HLC (P=0.940), Table 2A. Also, there was no significant interaction between presence of treatment and distance as a continuous variable on *Ae. albopictus* HLC (P=0.777), Table 2A. Factoring distance and controlling for environmental factors, there was a significant interaction between treatment and distance only at 5 m

(P=0.014), Table 2B. For the interaction of treatment and distances of 6m and 7m, ~~the~~ there was a marginally significant effect (P<0.06), Table 2B.

Table 2. IRR estimated from GLMM of landing counts upon exposure to emanators at different distances outdoors.

Model	Parameter	Value	Standard Error	Z-value	*P*-value
**A. Distance as a continuous variable**	Intercept	-1.74125	3.2169	-0.54	0.588
	Treatment (Control)	0.02895	0.38717	0.07	0.940
	Distance	0.05326	0.04745	1.12	0.262
	Humidity	0.03819	0.01908	2.00	**0.045**
	Temperature	0.00716	0.07698	0.09	0.926
	Wind Speed	0.07679	0.06897	1.11	0.266
	Treatment*Distance	0.1754	0.06188	0.28	0.777
**B. Distance as a factor**	Intercept	-0.6831	3.0854	-0.22	0.825
	Treatment (Control)	-0.3552	0.2913	-1.22	0.223
	Temperature	-0.0168	0.0735	-0.23	0.819
	Humidity	0.0373	0.0186	2.00	**0.045**
	Wind Speed	0.0679	0.0677	1.00	0.316
	Distance (4m)	0.1421	2.899	2.78	**0.0055**
	Distance (5 m)	-0.313	0.134	-2.33	**0.0199**
	Distance (6 m)	-0.1885	0.2601	-0.72	0.469
	Distance (7 m)	-0.2446	0.2637	-0.93	0.354
	Distance (8 m)	0.4664	0.2254	2.07	**0.039**
	Treatment*Distance (4 m)	0.2803	0.3506	0.80	0.424
	Treatment*Distance (5 m)	0.9479	0.3506	0.80	**0.014**
	Treatment*Distance (6 m)	0.6892	0.3634	1.90	0.058
	Treatment*Distance (7 m)	0.7085	0.3693	1.92	0.055
	Treatment*Distance (8 m)	0.0663	0.3373	0.20	0.844

In parenthesis is listed the variable used as baseline for effect estimation. Bolded p-values are less than 0.05 and statistically significant.

After factoring distance and controlling for environmental factors, there was a significant interaction between treatment and distance at 5 m (P=0.014), Table 2B. For distances of 6m and 7m, the effect of emanators was marginally significant (P<0.06), suggesting a potential effect, Table 2B. Of the environmental factors tested, there was a 1% increase in landing counts per percentage increase in humidity (P=0.045), Table 2B.

Study 3. Testing the effect of metofluthrin emanators on entry behavior

We recorded 436 landing counts over the course of four data collection days with 23.8% of all landings occurring in the presence of an emanator and 76.1% of all landings occurring during control periods. The mean landing count of the control was at least 1.7 times the mean landing count of the emanator (Fig 2C). Controlling for humidity, temperature, and wind speed, a GLMM showed metofluthrin emanators significantly reduced *Ae. albopictus* entry to the tent and subsequent landing with a 74.6% reduction in landing counts recorded in the presence of an emanator (P<0.001; Fig 2C), Table 3. Humidity, temperature, and wind speed were included in the model to reduce variance between days. Of the environmental factors tested, the model presented that there was a 7.5% increase in landing counts per one unit increase of wind speed in miles per hour (P=0.0046), Table 3.

Table 3. IRR estimated from a GLMM with parameters affecting mosquito premise entry and landing behavior.

**Parameter**	**Value**	**Standard Error**	***Z*-value**	***P*-value**	***1-IRR***
Intercept	-205.323	74.577	-2.75	**<0.001**	
Treatment (Emanator)	-1.369	0.323	-4.24	**<0.001**	0.746
Temperature	8.053	2.899	2.78	**0.0055**	
Humidity	-0.313	0.134	-2.33	**0.0199**	
Wind Speed	2.02	0.713	2.83	**0.0046**	

In parenthesis is listed the variable used as baseline for effect estimation. For treatments showing a significant reduction in HLC, we estimated the efficacy, as 1 - Incidence Rate Ratios (IRR). Bolded p-values are less than 0.05 and statistically significant.

Study 4. Testing emanator longevity and range of activity indoors

We recorded 2,885 HLC over the course of seven collection days with 42.8% of all landings occurring in the presence of an emanator and 57.2% occurring during control periods. Average landing counts during control periods was higher than in the presence of an emanator at both 1 and 3 m (Fig 3A). After accounting for distance and number of mosquitos, the time mosquitos were exposed to an emanator was a significant predictor of HLCs, Table 4A. After 30 minutes of exposure to the emanator, each additional minute of exposure led to a 2% reduction in mosquito landings (IRR=-0.02; P=0.013), Table 4A. HLCs were reduced at 60 minutes of exposure in comparison to 30 minutes of exposure (Fig 3B). A GLMM quantified that the emanator reduced landing counts by 61.1% in mosquitos that had been exposed for at least 60 minutes (P<0.001), Table 4B. The interaction variable of treatment and distance showed no significance (P=0.4114; Fig 3C), Table 4A.

Fig 3. Mean landing counts by time of exposure and distance from treatment indoors (Study 3).

(A) Emanator range of impact on *Ae. albopictus*. (B) Effect of emanator by time of exposure to emanator. (C) Effect of emanator by distance from treatment after 60 minutes of exposure.

Table 4. IRR estimates from a GLMM Model of mosquito landing counts upon exposure to metofluthrin emanators at different distances indoors.

**Model**	**Parameter**	**Value**	**Standard Error**	**Z-value**	***P*-value**	***1-IRR***
A. Determining impact of time on landing rates.	Intercept	-5.90479	0.74744	-7.90	**<0.001**	
	Treatment (Emanator)	0.77661	0.40381	1.92	0.054	
	Time	-0.00523	0.00594	-0.88	0.378	
	Distance (3 m)	1.91	0.12917	1.48	0.140	
	Treatment*Time	-0.02157	0.00872	-2.47	**0.013**	**0.02**
B. Assessing impact of distance on effect of treatment after 60 minutes of treatment exposure.	Intercept	-6.0017	0.6678	-8.99	**<0.001**	
	Treatment (Emanator)	-0.9452	0.2809	-3.36	**<0.001**	**0.611**
	Distance (3 m)	0.0127	0.2575	0.05	0.9607	
	Treatment*Distance	0.3109	0.3785	0.82	0.4114	

In parenthesis is listed the variable used as baseline for effect estimation. For treatments showing a significant reduction in HLC, we estimated the efficacy, as 1 - Incidence Rate Ratios (IRR). Bolded p-values are less than 0.05 and statistically significant.

Accounting for day and mosquito group, a CPHRM found that the probability of mortality among *Ae. albopictus* exposed to the emanator was 1.72 times higher than for *Ae. albopictus* exposed to the control in the control group (P=0.0120), Table 5. The impact of distance from an emanator on the probability of mosquito mortality was assessed on a

subset of the original data that included only mosquitos exposed to the emanator. Accounting for mosquito group and collection day, distance from the emanator (1 to 3 m) did not impact mosquito mortality (P=0.3672), Table 5.

Table 5. Estimates from a CPHM of parameters affecting mosquito mortality upon exposure to emanators indoors.

Parameter	Value	Standard Error	Z-value	*P*-value
Treatment (Control)	-0.5697	0.2268	-2.51	**0.0120**
Distance	-0.0518	0.05741	-0.90	0.3672
Treatment (Control)*Distance	0.2047	0.09815	2.09	**0.0370**

Day B is 11/10/17, Day C is 11/17/17, Day D is 11/25/17, Day E is 12/1/17, Day F is 12/9/17. In parenthesis is listed the variable used as baseline for effect estimation. Bolded p-values are less than 0.05 and statistically significant.

The 95% confidence interval of the prediction of the reduction in landings as emanator age increased led to an estimate maximum residual life of 2.5 weeks, with average reduction in HLCs of 1.5 weeks (Fig 4). Assessment of model fit confirmed that the correct smoothness parameter was selected with a statistically significant p-value (Landings~Age, F=4.766, P=0.0262).

Fig 4. Modeling the impact of emanator age on HLC. General additive model results from the trial testing range of effect of metofluthrin emanators on *Ae. albopictus* landing counts (*f(HLC)*) indoors compared to a trial without emanator. The zero line shows the no-effect threshold and dotted lines the 95% confidence interval of f(HLC). Anything above the line shows significant increase in HLCs compared to control, anything below the line shows the opposite.

No KDC were recorded at 30 minutes of emanator exposure. Thus, the first model assessing the treatment's impact on mosquito knock downs included mosquitoes exposed to the treatments after 60 minutes. The emanator significantly knocked down *Ae. albopictus* mosquitoes (P<0.001), Table 5. This result was consistent with data collection as no knock downs occurred without the emanator. Distance from the emanator did not significantly impact the likelihood of an *Ae.* ~~*aegypti*~~ mosquito being knocked down (P=0.4233).

Accounting for day and mosquito group, a CPHRM found that the probability of mortality among *Ae. albopictus* exposed to the emanator was 1.72 times higher than for *Ae. albopictus* exposed to the control in the control group (P=0.0120), Table 6. The impact of distance from an emanator on the probability of mortality was assessed on a subset of the original data that included only mosquitoes exposed to the emanator. Accounting for mosquito group and collection day, distance from the emanator (1 to 3 m) did not impact mosquito mortality (P=0.3672), Table 6.

Table 6. Parameters from Cox Proportional Hazards Regression Model affecting *Aedes albopictus* mortality post-exposure to emanators indoors.

Parameter	Value	Standard Error	Z-value	*P*-value
Treatment (Control)	-0.5697	0.2268	-2.51	**0.0120**
Distance	-0.0518	0.05741	-0.90	0.3672
Treatment (Control)*Distance	0.2047	0.09815	2.09	**0.0370**

The variable used as baseline for effect estimation is listed in parenthesis. Bolded p-values are less than 0.05 and statistically significant.

The 95% confidence interval of the prediction of the reduction in landings as emanator age increased led to an estimate maximum residual life of 2.5 weeks, with average reduction of 1.5 weeks (Fig 4A). Assessment of model fit confirmed that the correct smoothness parameter was selected with a statistically significant p-value (Landings~Age, F=4.766, P=0.0262). Knock-down was similarly affected, with 95% confidence intervals predicting a maximum residual effect up to 3.8 weeks and an average effect of 3.1 weeks (Fig 4B). Model fit was confirmed by a statistically significant p-value (Knock Downs ~ Age, F=4.897, P=0.00566).

~~**Fig 4. Modeling the impact of emanator age on HLC.**~~

~~General additive model results from the trial testing range of effect of metofluthrin emanators on *Ae. albopictus* landing counts (*f(HLC)*) indoors compared to a trial without emanator. The zero line shows the no-effect threshold and dotted lines the 95% confidence interval of f(HLC). Anything above the line shows significant increase in HLCs compared to control, anything below the line shows the opposite.~~

Discussion

Extending findings observed for *Ae. aegypti* [33] we show that a 10% metofluthrin passive emanator has high potential to reduce human-*Ae. albopictus* contacts both indoors and outdoors. Our study expands upon laboratory evaluations of metofluthrin passive emanators against *Ae. albopictus* by conducting studies under more realistic indoor and

outdoor settings with a wild mosquito population that showed susceptibility to all insecticide classes.

In most settings, and particularly in the US, *Ae. albopictus* is a peridomestic vector that aggressively bites humans and other vertebrate hosts outdoors. In Atlanta and nearby locations, *Ae. albopictus* is the dominant mosquito species in residential areas [39] very often reaching high densities. As observed in our study, it is very common for observers to experience more than 100 landings in 5 minutes of outdoor exposure. Application of barrier insecticide sprays in low vegetation within yards alone or in combination with larval control has been shown to be effective in reducing *Ae. albopictus* density [40]. Unfortunately, the high costs and large environmental impacts (particularly on non-target species) of widespread barrier spraying in residential areas may limit its scalability in highly urbanized regions of the US and Europe. In areas prone to *Ae. albopictus* invasion or at first detection, such approaches may be justified [41]. However, areas like Atlanta, where *Ae. albopictus* is fully established, such barrier insecticide sprays may not be feasible beyond privately contracted pest control services.

Minimizing human-mosquito contacts using passive metofluthrin emanators may provide an environmentally friendly personal protection alternative to reduce the nuisance and risk of *Ae. albopictus*. Our study shows over 90% reduction in landings when a 10% metofluthrin emanator is placed outdoors next to a human volunteer. Furthermore, while marginal reductions were observed at increasing distances of the emanator (up to 7 meters), the efficacy of the product may be increased by having a product with higher % of a.i. (the prototype we used was a 10% w/w formulation but alternatives with higher a.i. might be engineered). Further, commercial metofluthrin products exist (e.g., Off! Clip-on, an active emanator, SJ Johnsons, Racine, WI) and their efficacy against *Ae. albopictus* was slightly

lower when worn by human volunteers outdoors (70% reduction, versus 90% reduction in our study) [42]. In situation where people may be sitting outdoors (patios, outside tables in restaurants, porches) passive metofluthrin emanators may provide important protection with minimal insecticide use. The efficacy and range of possible applications of metofluthrin passive emanators remains to be studied.

Product longevity is key for the proper adoption of an intervention. For *Ae. aegypti*, the same product we evaluated provided protection for up to 21 days in experimental rooms [32]. We found that for *Ae. albopictus* metofluthrin emanators significantly reduced HLCs indoors up to 2.5 weeks from deployment. As an important fraction of the a.i. remains in the polyacrylamide mesh [29], novel engineering approaches may lead to longer duration of the emanator efficacy if they can minimize a.i. retention by the mesh. Furthermore, compared to metofluthrin coils, passive emanators have significantly longer duration and the added benefit of not requiring burning or other source of heat. In Merida, Mexico, household surveys showed that people view very positively the use of metofluthrin emanators as alternative to coils and reported measurable reductions in mosquito bites after their use [33].

Previous research determined that ten minutes of exposure indoors reduced *Ae. aegypti* landing counts [32, 26]. Buhagiar et al 2017 found that the devices' effectiveness increases the longer it is implemented likely due to the slow dispersal of the molecule into an environment [26]. The reduction in landings we observed at one hour coincided with an increased number of mosquitoes exhibiting intoxication signs such as paralysis. Such effect was larger after 2 hours of exposure. Therefore, implementation guidelines should recommend the device be hung in an indoor ventilated location for at least one hour to ensure high protective efficacy to those entering or residing in it.

There were several limitations in our study. In outdoor experiments, while we controlled for environmental conditions in our analysis (temperature, humidity, wind) we could not sustain similar environmental conditions, which may explain why some days had different reductions in landings than others. Repeating the experiment for more days would have shown stronger effects, particularly when evaluating the influence of emanators at increasing distances outdoors. The marginal associations found at 6-7 m may be influence of prevailing wind or other factors, and the experiment should be repeated to ensure the actual range of efficacy outdoors. Unfortunately, limited number of emanators prevented us from repeating that study. Also, the time the emanator was evaluated outdoors (5 min) may have not been sufficient. A study evaluating metofluthrin active emanator Off! Clip-on evaluated efficacy over a 3h period [42]. Extending beyond 5 minutes may have provided a temporal signal of the effect of metofluthrin on mosquito attraction, biting and repellency. Metofluthrin not only repels mosquitos but also works as a “confusant” with additional lethal impacts [32]. In our trial the behavioral impacts of metofluthrin (which were landing counts and reduced entry to tents) were evident after 60 minutes, but future experiments should determine if mosquitoes repelled by the device are later knocked down.

In our current study *Ae. albopictus* exposed to a 10% metofluthrin emanator indoors reduced landings by 70% regardless of the distance to the emanator within a regular bedroom (3m), showing potent efficacy as reported in other studies. A recent entomological randomized field trial of the same emanator prototype in Merida, Mexico, showed that when placed at a rate of one per bedroom, emanators reduced indoor *Ae. aegypti* abundance by 70% [33]. Our current experimental findings coupled with the field trial results for *Ae. aegypti* [33] provide enough evidence to support the design of further trials for *Ae.*

albopictus. The rapid invasion of *Ae. albopictus* in Europe, with subsequent recent occurrence of arbovirus outbreaks [17] requires vector control methods that can be effective and scalable. If efficacious, metofluthrin emanators could be deployed in affected areas (for instance, during outbreaks) for rapid reduction of human-mosquito contacts and disease. Provided mosquitoes are susceptible to the a.i., passive emanators can fill an important niche in urban control of *Aedes*-transmitted arbovirus.

Acknowledgment

We would like to thank ~~to~~ graduate and undergraduate students from the Department of Environmental sciences, Emory University, for their collaboration in data collection.

References

1. Roiz D, Wilson AL, Scott TW, Fonseca DM, Jourdain F, Muller P, et al. Integrated Aedes management for the control of Aedes-borne diseases. PLoS Negl Trop Dis. 2018;12(12):e0006845.
2. San Martin JL, Brathwaite O, Zambrano B, Solorzano JO, Bouckenooghe A, Dayan GH, et al. The epidemiology of dengue in the americas over the last three decades: a worrisome reality. Am J Trop Med Hyg. 2010;82(1):128-35.

3. Paixao ES, Teixeira MG, Rodrigues LC. Zika, chikungunya and dengue: the causes and threats of new and re-emerging arboviral diseases. BMJ Glob Health. 2018;3(Suppl1):e000530.

4. Kilpatrick AM, Randolph SE. Drivers, dynamics, and control of emerging vector-borne zoonotic diseases. Lancet. 2012;380(9857):1946-55.

5. Center for Disease Control. Surveillance and Control of Aedes aegypti and Aedes albopictus in the United States. 2017.

6. Jentes ES, Lash RR, Johansson MA, Sharp TM, Henry R, Brady OJ, et al. Evidence-based risk assessment and communication: a new global dengue-risk map for travelers and clinicians. J Travel Med. 2016;23(6).

7. Gibney KB, Fischer M, Prince HE, Kramer LD, St George K, Kosoy OL, et al. Chikungunya fever in the United States: a fifteen year review of cases. Clin Infect Dis. 2011;52(5):e121-6.

8. Hennessey MJ, Fischer M, Panella AJ, Kosoy OI, Laven JJ, Lanciotti RS, et al. Zika Virus Disease in Travelers Returning to the United States, 2010-2014. Am J Trop Med Hyg. 2016;95(1):212-5.

9. Center for Disease Control. Locally acquired dengue-key west, Florida, 2009–2010. Morb Mortal Wkly. 2010;Rep 59:577-81.

10. Brunkard JM, Robles Lopez JL, Ramirez J, Cifuentes E, Rothenberg SJ, Hunsperger EA, et al. Dengue fever seroprevalence and risk factors, Texas-Mexico border, 2004. Emerg Infect Dis. 2007;13(10):1477-83.

11. Ramos MM, Mohammed H, Zielinski-Gutierrez E, Hayden MH, Lopez JL, Fournier M, et al. Epidemic dengue and dengue hemorrhagic fever at the Texas-Mexico

border: results of a household-based seroepidemiologic survey, December 2005. Am J Trop Med Hyg. 2008;78(3):364-9.

12. Center for Disease Control. Chikungunya Virus in the United States. 2018.

13. Graham AS, Pruszynski CA, Hribar LJ, DeMay DJ, Tambasco AN, Hartley AE, et al. Mosquito-associated dengue virus, Key West, Florida, USA, 2010. Emerg Infect Dis. 2011;17(11):2074-5..

14. Parker C, Ramirez D, Connelly CR. State-wide survey of Aedes aegypti and Aedes albopictus (Diptera: Culicidae) in Florida. J Vector Ecol. 2019;44(2):210-5.

15. Richards SL, Anderson SL, Alto BW. Vector competence of Aedes aegypti and Aedes albopictus (Diptera: Culicidae) for dengue virus in the Florida Keys. J Med Entomol. 2012;49(4):942-6.

16. Lounibos LP, Bargielowski I, Carrasquilla MC, Nishimura N. Coexistence of Aedes aegypti and Aedes albopictus (Diptera: Culicidae) in Peninsular Florida Two Decades After Competitive Displacements. J Med Entomol. 2016;53(6):1385-90.

17. Schaffner F, Mathis A. Dengue and dengue vectors in the WHO European region: past, present, and scenarios for the future. Lancet Infect Dis. 2014;14(12):1271-80.

18. Benedict MQ, Levine RS, Hawley WA, Lounibos LP. Spread of the tiger: global risk of invasion by the mosquito Aedes albopictus. Vector Borne Zoonotic Dis. 2007;7(1):76-85.

19. Swan T, Lounibos LP, Nishimura N. Comparative Oviposition Site Selection in Containers by Aedes aegypti and Aedes albopictus (Diptera: Culicidae) from Florida. J Med Entomol. 2018;55(4):795-800.

20. Braks MA, Honorio NA, Lourencqo-De-Oliveira R, Juliano SA, Lounibos LP. Convergent habitat segregation of Aedes aegypti and Aedes albopictus (Diptera: Culicidae) in southeastern Brazil and Florida. J Med Entomol. 2003;40(6):785-94.
21. Lounibos LP, Kramer LD. Invasiveness of Aedes aegypti and Aedes albopictus and Vectorial Capacity for Chikungunya Virus. J Infect Dis. 2016;214(suppl 5):S453-S8.
22. Garcia-Luna SM, Weger-Lucarelli J, Ruckert C, Murrieta RA, Young MC, Byas AD, et al. Variation in competence for ZIKV transmission by Aedes aegypti and Aedes albopictus in Mexico. PLoS Negl Trop Dis. 2018;12(7):e0006599.
23. Ciota AT, Bialosuknia SM, Zink SD, Brecher M, Ehrbar DJ, Morrissette MN, et al. Effects of Zika Virus Strain and Aedes Mosquito Species on Vector Competence. Emerg Infect Dis. 2017;23(7):1110-7.
24. Vega-Rúa A, Zouache K, Girod R, Failloux AB, Lourenço-de-Oliveira R. High level of vector competence of Aedes aegypti and Aedes albopictus from ten American countries as a crucial factor in the spread of Chikungunya virus. J Virol. 2014;88(11):6294-306.
25. Muzari MO, Devine G, Davis J, Crunkhorn B, van den Hurk A, Whelan P, et al. Holding back the tiger: Successful control program protects Australia from Aedes albopictus expansion. PLoS Negl Trop Dis. 2017;11(2):e0005286.
26. Buhagiar TS, Devine GJ, Ritchie SA. Metofluthrin: investigations into the use of a volatile spatial pyrethroid in a global spread of dengue, chikungunya and Zika viruses. Parasites & Vectors. 2017;10(1):270.

27. Achee NL, Bangs MJ, Farlow R, Killeen GF, Lindsay S, Logan JG, et al. Spatial repellents: from discovery and development to evidence-based validation. Malar J. 2012;11:164.

28. Bibbs CS, Tsikolia M, Bloomquist JR, Bernier UR, Xue RD, Kaufman PE. Vapor toxicity of five volatile pyrethroids against Aedes aegypti, Aedes albopictus, Culex quinquefasciatus, and Anopheles quadrimaculatus (Diptera: Culicidae). Pest Manag Sci. 2018;74(12):2699-706.

29. Matsuo N, Ujihara K, Shono Y, Iwasaki T, Sugano M, Yoshiyama T, et al. Discovery and Development of a Novel Pyrethroid Insecticide 'Metofluthrin (SumiOne®, Eminence®)'. Sumitomo Kagaku: Sumitomo Chemical Co., Ltd.; 2005.

30. Chen CD, Chin AC, Lau KW, Low VL, Lee HL, Lee PKY, et al. Bioefficacy Evaluation of Commercial Mosquito Coils Containing Metofluthrin, d-Allethrin, d-Trans Allethrin, and Prallethrin Against Aedes albopictus (Diptera: Culicidae) in Malaysia. J Med Entomol. 2018.

31. Ujihara K, Matsuo N, Mori T, Shono Y, Iwasaki T. Metofluthrin: Novel Pyrethroid Insecticide and Innovative Mosquito Control Agent. J PESTIC SCI. 2007;33(2).

32. Ritchie SA, Devine GJJP, Vectors. Confusion, knock-down and kill of Aedes aegypti using metofluthrin in domestic settings: a powerful tool to prevent dengue transmission? 2013;6(1):262.

33. Devine GJ, Vazquez-Prokopec GM, Bibiano-Marin W, Pavia-Ruz N, Che-Mendoza A, Medina-Barreiro A, et al. The entomological impact of passive metofluthrin emanators against indoor Aedes aegypti: A randomized field trial. PLoS Negl Trop Dis. 2021;15(1):e0009036.

34. World Health Organization. Guidelines for efficacy testing of mosquito repellents for human skin. Geneva: World Health Organization., Diseases CoNT; 2008.

35. World Health Organization. Guidelines for Efficacy Testing of Spatial Repellents. Diseases CoNT; 2013.

36. World Health Organization. Monitoring and managing insecticide resistance in *Aedes* mosquito populations. Interim guidance for entomologists. Geneva: World Health Organization, Programme CoNTDaGM; 2016.

37. Darbro JM, Muzari MO, Giblin A, Adamczyk RM, Ritchie SA, Devine GJ. Reducing biting rates of Aedes aegypti with metofluthrin: investigations in time and space. Parasit Vectors. 2017;10(1):69.

38. Brogdon WG, Chan, A. Guideline for Evaluating Insecticide Resistance in Vectors Using the CDC Bottle Bioasssay (with Inserts 1 in 2012 and 2 in 2014). 2010.

39. Evans MV, Hintz CW, Jones L, Shiau J, Solano N, Drake JM, et al. Microclimate and Larval Habitat Density Predict Adult Aedes albopictus Abundance in Urban Areas. Am J Trop Med Hyg. 2019;101(2):362-70.

40. Hollingsworth B, Hawkins P, Lloyd AL, Reiskind MH. Efficacy and Spatial Extent of Yard-Scale Control of Aedes (Stegomyia) albopictus (Diptera: Culicidae) Using Barrier Sprays and Larval Habitat Management. J Med Entomol. 2020;57(4):1104-1

41. Baldacchino F, Caputo B, Chandre F, Drago A, della Torre A, Montarsi F, et al. Control methods against invasive Aedes mosquitoes in Europe: a review. Pest Manag Sci. 2015;71(11):1471-85.

42. Xue RD, Qualls WA, Smith ML, Gaines MK, Weaver JH, Debboun M. Field evaluation of the Off! Clip-on Mosquito Repellent (metofluthrin) against Aedes

albopictus and Aedes taeniorhynchus (Diptera: Culicidae) in northeastern Florida. J Med Entomol. 2012;49(3):652-5.

Supporting information

S1 Table. Final Generalized Linear Mixed Models by Outdoor Trials. Impact of treatment outdoors on the response variables: landings and knock downs.

Null Hypothesis	Model Type	Distribution	Response Variable	Fixed Effects	Random Effects
The metofluthrin emanator does not impact *Aedes albopictus* feeding behavior outdoors.	GLMM[a]	Negative Binomial	Landings	Treatment	Day
There is no difference in *Aedes albopictus* feeding behavior at 3-8 meters from a 10% metofluthrin emanator outdoors.	GLMM[a]	Zero-inflated Negative Binomial	Landings	Treatment*Distance, Temperature, Humidity, Wind Speed<br><br>Treatment*factored(Distance), Person, Temperature, Humidity, Wind Speed	Day/Trial, Person<br><br>Day/Trial
The age of the metofluthrin emanator does not impact *Aedes albopictus* feeding behavior outdoors.	GAM[b]		Landings	Emanator Age	

[a]GLMM = Generalized Linear Mixed Model

[b]GAM = Generalized Additive Model

S2 Table. Final Generalized Linear Mixed Models by Indoor Trials. Impact of treatment indoors on the response variables: landings and knock downs.

Null Hypothesis	Model Type	Distributio n	Response Variable	Fixed Effects	Rando m Effects
The 10% metofluthrin emanator does not impact *Aedes albopictus* feeding behavior indoors.	GLMM[a]	Negative Binomial	Landings	Treatment, Temperature, Humidity, Wind Speed	Day/ Trial
There is no difference in *Aedes albopictus* feeding behavior at 1-3 meters from a metofluthrin emanator indoors	GLMM[a]	Negative Binomial	Landings	Treatment*Time + Distance + Number of Mosquitoes<br><br>Treatment*Distance + Number of Mosquitoes	Day, Person<br><br>Day, Person
		Binomial	Knock downs	Treatment<br><br>Distance	Day<br><br>Day
The metofluthrin emanator does not impact *Aedes albopictus* mortality.	CPHRM[b]		Mortality	Treatment*factored(Distance) + cluster(Group) + Day<br><br>Distance + cluster(Group) + Day	
The age of the metofluthrin emanator does not impact *Aedes albopictus* feeding behavior indoors.	GAM[c]		Landings<br><br>Knock downs	Emanator Age<br><br>Emanator Age	

The model assessing the effect of distance from the emanator on mosquito landing used a subset of the original data which included only landings from mosquitoes exposed to a treatment for 60 minutes. Models assessing the effect of distance from the emanator on mosquito knock downs and mortality used a subset of the original data which included only mosquitoes exposed to the emanator.

[a]GLMM = Generalized Linear Mixed Model

[b]CPHR = Cox Proportional Hazards Regression Model

[c]GAM = Generalized Additive Model

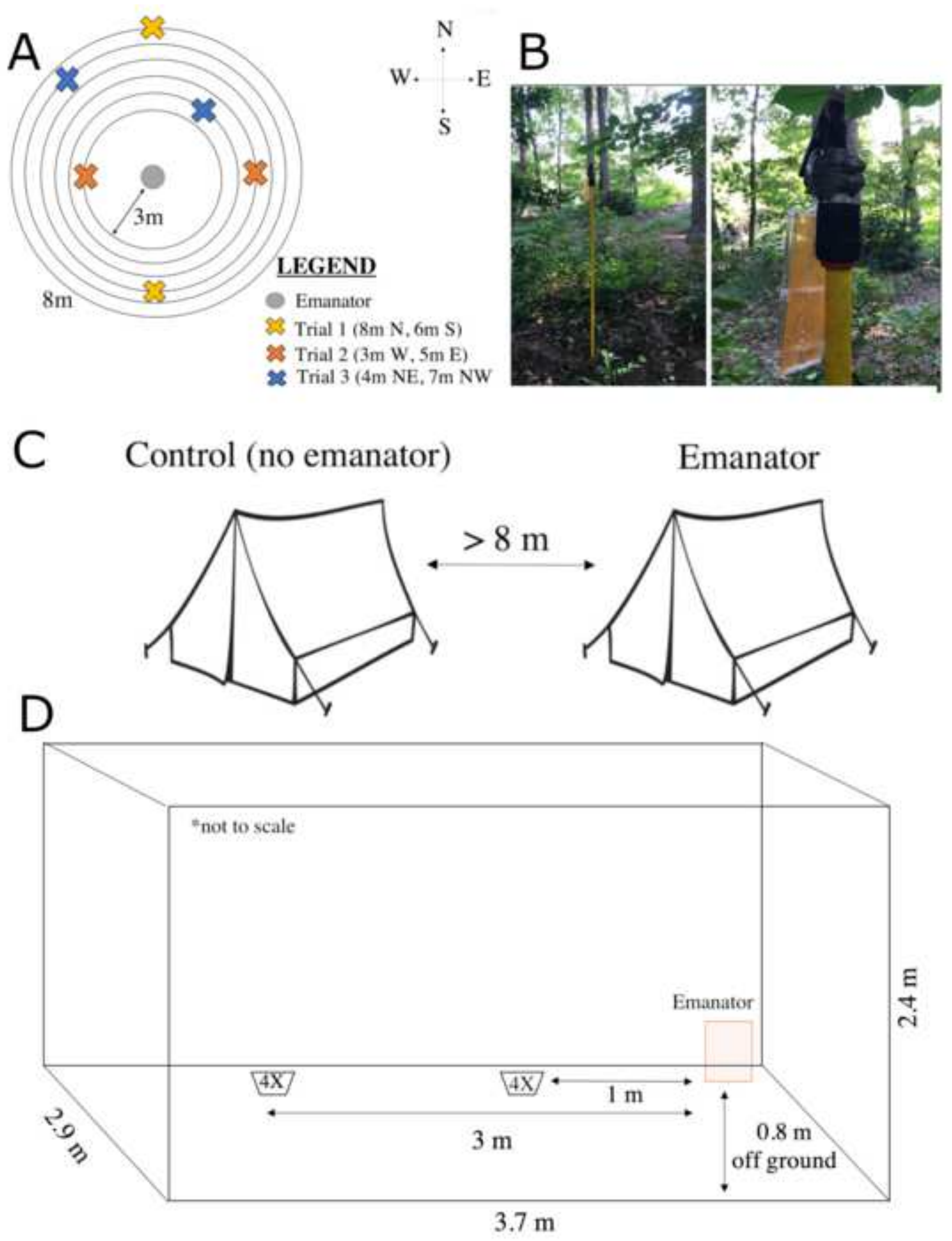
A
N
W
E
S
3m
8m
LEGEND
Emanator
Trial 1 (8m N, 6m S)
Trial 2 (3m W, 5m E)
Trial 3 (4m NE, 7m NW
B
C
Control (no emanator)
Emanator
> 8 m
D
*not to scale
Emanator
4X
4X
1 m
3 m
0.8 m
off ground
2.4 m
2.9 m
3.7 m

Figure 2

Click here to access/download;Figure;Fig2.png

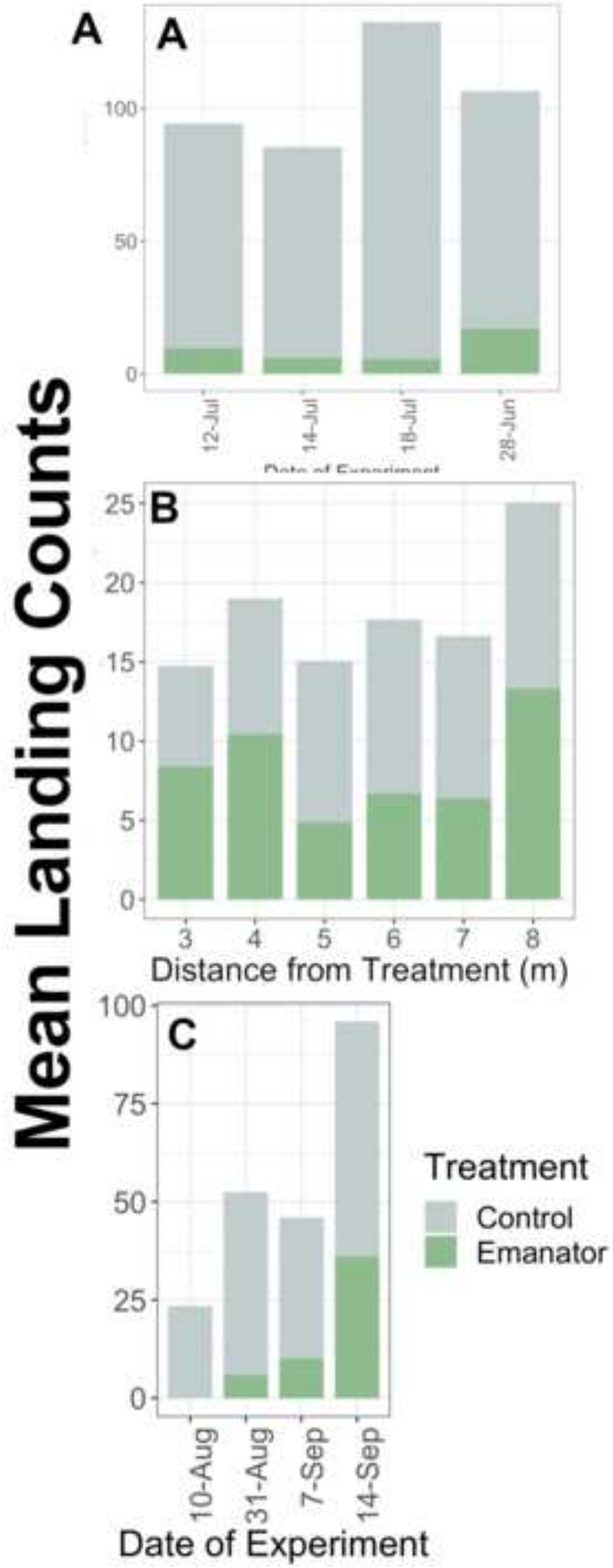

Figure 3

Click here to access/download;Figure;Fig3.png

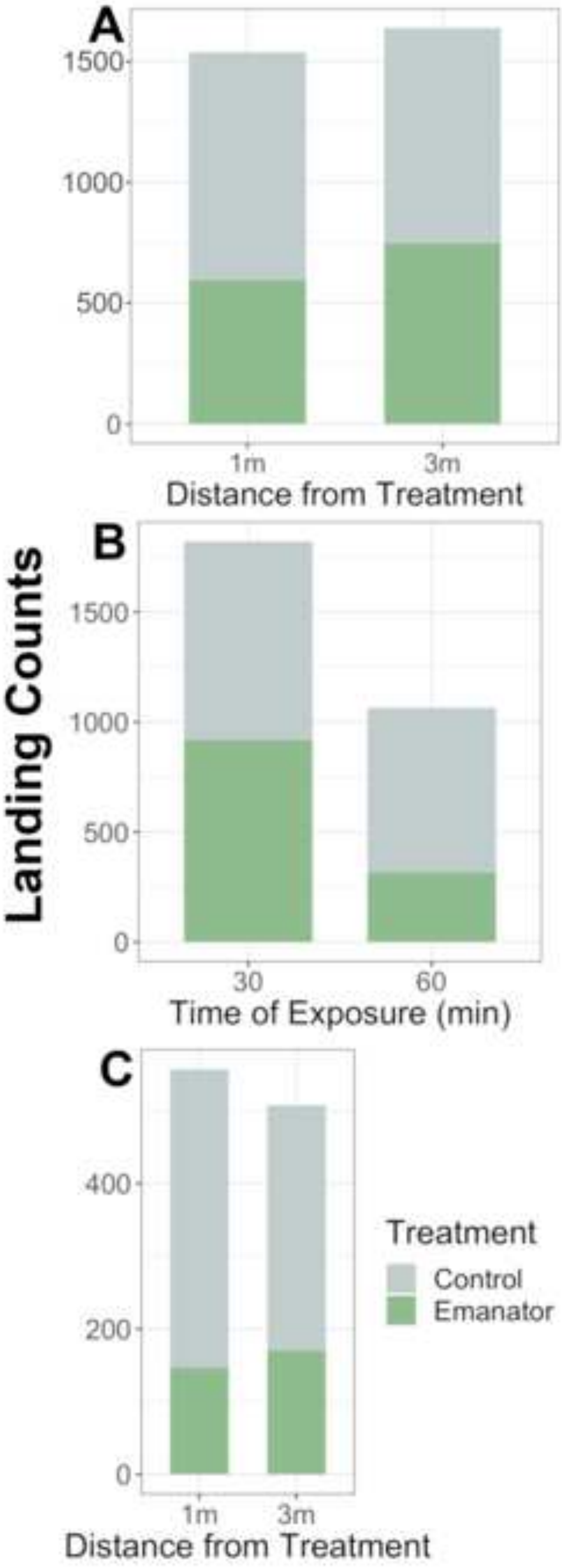

Figure 4

Click here to access/download;Figure;Figure4.tif

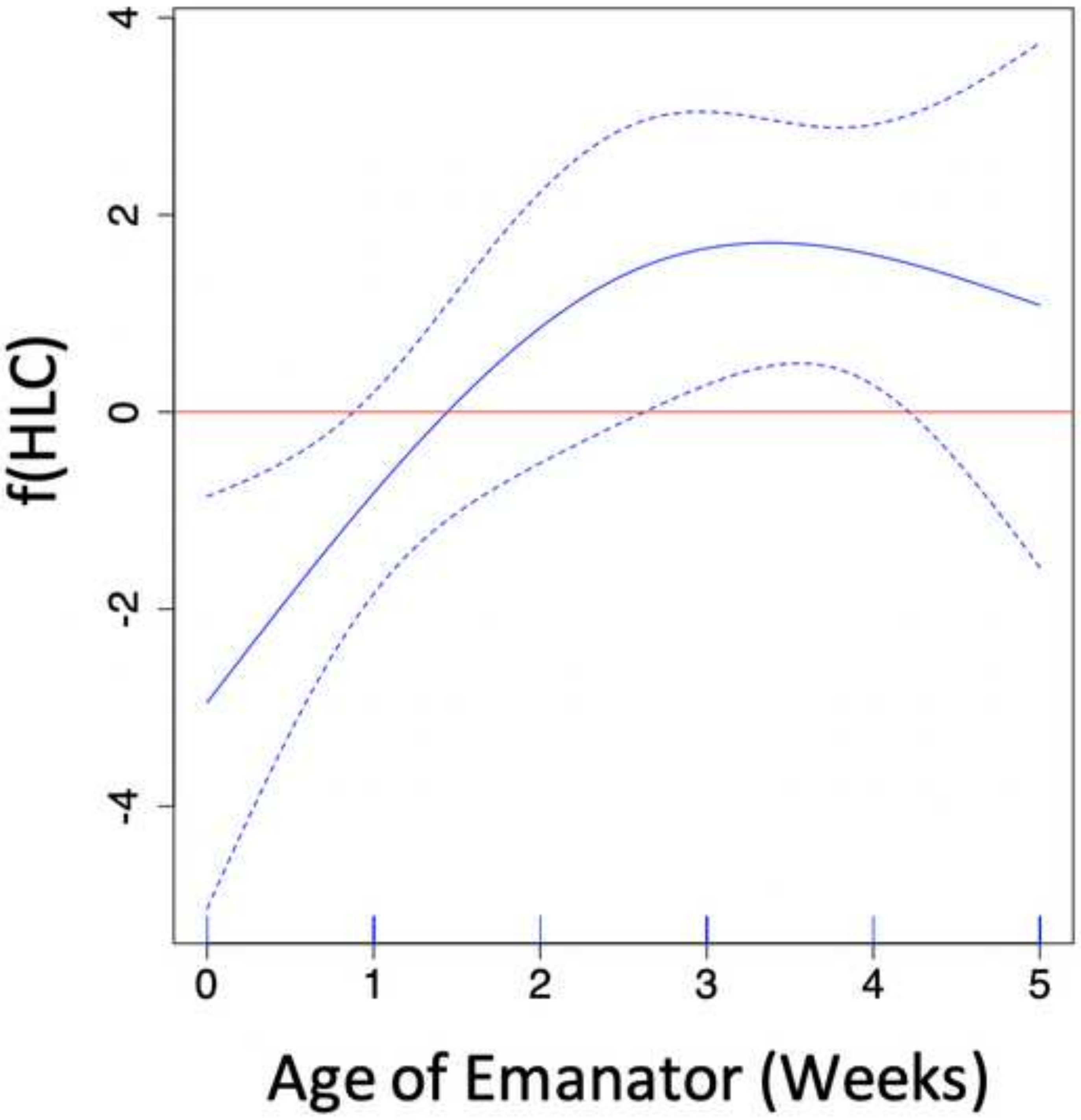

Supplement: Attachment — Submitted filename: PONE-D-21-25159 (3)reviewed.pdf [file pone.0267278.s002.pdf]
