# Supplementary material for: Experimental evaluation of a metofluthrin passive emanator against Aedes albopictus
Source: PLoS One. 2022 May 10;17(5):e0267278. doi: 10.1371/journal.pone.0267278 (PMC9089854; doi:10.1371/journal.pone.0267278)
Supplement: Attachment — Submitted filename: Response to Reviewers.docx [file pone.0267278.s003.docx]

PLoS ONE

Editorial Office

Thank you for providing feedback on our manuscript submitted to PLOS ONE. We have addressed all the reviewers’ and editor concerns and provide a revised draft of our manuscript for your consideration.

We enclose the following:

- A rebuttal letter that responds to each point raised by the academic editor and reviewer(s). You should upload this letter as a separate file labeled 'Response to Reviewers'.
- A marked-up copy of your manuscript that highlights changes made to the original version. You should upload this as a separate file labeled 'Revised Manuscript with Track Changes'.
- An unmarked version of your revised paper without tracked changes. You should upload this as a separate file labeled 'Manuscript'.

Best regards,

Gonzalo Vazquez Prokopec

====================================================================================

ACADEMIC EDITOR: This study includes human in the experiment, so, ethical approve should be included in the manuscript.
**R: We have obtained IRB approval for this study, which now is listed as a statement as follows “Methods for landing counts were approved by Emory University IRB (IRB00082773: Backyard Sampling of *Aedes* albopictus protocol). Written consent was obtained from all participants”.**

EDITORIAL REQUIREMENTS:

1. Please ensure that your manuscript meets PLOS ONE's style requirements, including those for file naming. The PLOS ONE style templates can be found at

**R: DONE**

2. We note that you have stated that you will provide repository information for your data at acceptance. Should your manuscript be accepted for publication, we will hold it until you provide the relevant accession numbers or DOIs necessary to access your data. If you wish to make changes to your Data Availability statement, please describe these changes in your cover letter and we will update your Data Availability statement to reflect the information you provide.

**R. Done. Data are available from Mendeley Data.**

**Vazquez-Prokopec, Gonzalo (2021), “Experimental evaluation of a metofluthrin passive emanator against Aedes albopictus”, Mendeley Data, V1, doi: 10.17632/dkdc8642cv.1**

3. Please note that supplementary tables should be uploaded as separate "supporting information" files"

**R. Done**

**Reviewers' comments:**
Reviewer #1: It is a comprehensive study on metofluthion against Aedes mosquitoes even if there are so many studies and literatures. This report provided useful information. However, I did not find the provided number about using human subjects for the landing rate counts and the approved animal care number for the blood feeding for the lab colony.
Bibbs CS did several years study about the same compound and published several important articles should be checked and cited. J. Med. Entomol. 53:480-487,2016, 57:17-24, 2020, and Environ Entomol. 49:435-443.
Khater EIM et al. 2021. Insecticide efficacy of spatial repellent compound metofluthrin against susceptible and resistant strains of Aedes aegypti. Journal of the Florida Mosquito Control Association 68:86-91. DOI.org/10.32473/JFMCA.v68i1.129104

**R. Thank you for the references. Additionally, we provide Emory’s IRB code, now reviewed and approved by the IRB. Per Emory University procedures, we are not required to obtain approval to use heparinized rabbit blood for feeding laboratory-reared mosquitoes. Our lab has all approvals from Environmental Health Office required for mosquito rearing. No IACUC is required in that case.**

Reviewer #2: The paper is well written. It provided background information on the efficacy of metofluthrin-based devices on mosquito and the knowledge gap regarding metofluthrin-based emanators on Aedes albopictus. The findings of the study provide valuable information for personal protection against Ae. albopictus using the emanators. The limitations of the study provide guidance in interpreting and using the information generated as well as pointers for further studies.
A few repetitions were noted in the manuscript and these have been indicated for the authors to address. Other minor comments/corrections have been indicated. The x-axis caption of Fig. 2A is not legible.
**R. We have taken them all into consideration. Thank you.**

Of concern is the absence of ethical clearance for this study since it involved humans. In Human Landing Catch (HLC) experiments, human volunteers are used as baits and hence there is the need for ethical clearance for such studies. The authors should indicate why ethical clearance was not sought if that is the case.

**R. We have obtained ethical clearance and now we list it in the method section.**

6. PLOS authors have the option to publish the peer review history of their article ([what does this mean?](https://nam11.safelinks.protection.outlook.com/?url=https%3A%2F%2Fjournals.plos.org%2Fplosone%2Fs%2Feditorial-and-peer-review-process%23loc-peer-review-history&data=04%7C01%7Cgmvazqu%40emory.edu%7C3366fa553b7c46cbff2508d9836f2c48%7Ce004fb9cb0a4424fbcd0322606d5df38%7C0%7C0%7C637685336883582851%7CUnknown%7CTWFpbGZsb3d8eyJWIjoiMC4wLjAwMDAiLCJQIjoiV2luMzIiLCJBTiI6Ik1haWwiLCJXVCI6Mn0%3D%7C3000&sdata=b2O5KhWZWJIv%2BCi0W3hUp3ZjLJ4XofYSf32GpwMGPhg%3D&reserved=0)). If published, this will include your full peer review and any attached files.

===============================================================================

While revising your submission, please upload your figure files to the Preflight Analysis and Conversion Engine (PACE) digital diagnostic tool, [https://pacev2.apexcovantage.com/](https://nam11.safelinks.protection.outlook.com/?url=https%3A%2F%2Fpacev2.apexcovantage.com%2F&data=04%7C01%7Cgmvazqu%40emory.edu%7C3366fa553b7c46cbff2508d9836f2c48%7Ce004fb9cb0a4424fbcd0322606d5df38%7C0%7C0%7C637685336883592843%7CUnknown%7CTWFpbGZsb3d8eyJWIjoiMC4wLjAwMDAiLCJQIjoiV2luMzIiLCJBTiI6Ik1haWwiLCJXVCI6Mn0%3D%7C3000&sdata=nKjYsUjg2lXAJJoybENpHf893rNVafr7M8q1N%2FrgPN8%3D&reserved=0). PACE helps ensure that figures meet PLOS requirements. To use PACE, you must first register as a user. Registration is free. Then, login and navigate to the UPLOAD tab, where you will find detailed instructions on how to use the tool. If you encounter any issues or have any questions when using PACE, please email PLOS at [figures@plos.org](mailto:figures@plos.org). Please note that Supporting Information files do not need this step.

**R. Done.**
